# Underground metabolism facilitates the evolution of novel pathways for vitamin B6 biosynthesis

**DOI:** 10.1007/s00253-021-11199-w

**Published:** 2021-03-04

**Authors:** Björn Richts, Fabian M. Commichau

**Affiliations:** 1 Department of General Microbiology, Institute for Microbiology and Genetics, University of Goettingen, Grisebachstrasse 8, 37077 Göttingen, Germany; 2 FG Synthetic Microbiology, Institute for Biotechnology, BTU Cottbus-Senftenberg, Universitätsplatz 1, 01968 Senftenberg, Germany

**Keywords:** Pyridoxal 5′-phosphate, Promiscuous enzyme, Enzyme promiscuity, Genetic suppression, Enzyme specificity

## Abstract

**Abstract:**

The term vitamin B6 is a designation for the vitamers pyridoxal, pyridoxamine, pyridoxine and the respective phosphate esters pyridoxal-5′-phosphate (PLP), pyridoxamine-5′-phosphate and pyridoxine-5′-phosphate. Animals and humans are unable to synthesise vitamin B6. These organisms have to take up vitamin B6 with their diet. Therefore, vitamin B6 is of commercial interest as a food additive and for applications in the pharmaceutical industry. As yet, two naturally occurring routes for de novo synthesis of PLP are known. Both routes have been genetically engineered to obtain bacteria overproducing vitamin B6. Still, major genetic engineering efforts using the existing pathways are required for developing fermentation processes that could outcompete the chemical synthesis of vitamin B6. Recent suppressor screens using mutants of the Gram-negative and Gram-positive model bacteria *Escherichia coli* and *Bacillus subtilis*, respectively, carrying mutations in the native pathways or heterologous genes uncovered novel routes for PLP biosynthesis. These pathways consist of promiscuous enzymes and enzymes that are already involved in vitamin B6 biosynthesis. Thus, *E. coli* and *B. subtilis* contain multiple promiscuous enzymes causing a so-called underground metabolism allowing the bacteria to bypass disrupted vitamin B6 biosynthetic pathways. The suppressor screens also show the genomic plasticity of the bacteria to suppress a genetic lesion. We discuss the potential of the serendipitous pathways to serve as a starting point for the development of bacteria overproducing vitamin B6.

**Key points:**

*• Known vitamin B6 routes have been genetically engineered.*

*• Underground metabolism facilitates the emergence of novel vitamin B6 biosynthetic pathways.*

*• These pathways may be suitable to engineer bacteria overproducing vitamin B6.*

## Introduction

**Fig. 1:**
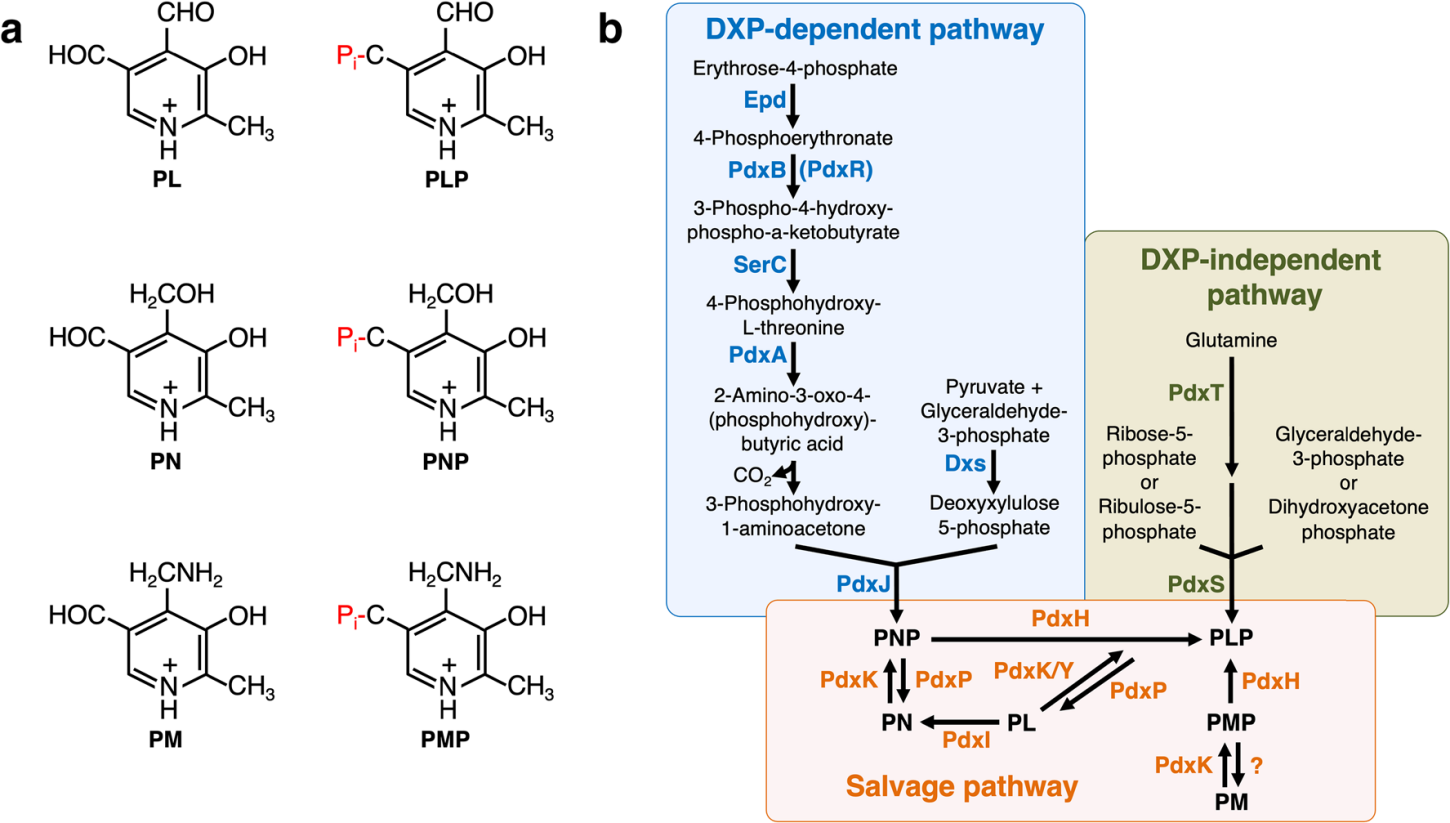
**a** The B6 vitamers pyridoxal (PL), pyridoxine (PN), pyridoxamine (PM) and the respective phosphate esters pyridoxal-5′-phosphate (PLP), pyridoxine-5′-phosphate (PNP) and pyridoxamine-5′-phosphate (PMP). **b** The deoxyxylulose 5-phosphate (DXP)-dependent and DXP-independent vitamin B6 biosynthetic routes and the salvage pathway for the interconversion of the B6 vitamers. Epd, erythrose 4-phosphate dehydrogenase; PdxB, 4-phosphoerythronate dehydrogenase; SerC, 3-phosphoserine aminotransferase; PdxA, 4-phosphohydroxy-l-threonine dehydrogenase; PdxJ, PNP synthase; Dxs, 1-deoxyxylulose 5-phosphate synthase; PdxH, PNP oxidase; PdxS (PLP synthase subunit) and PdxT (glutaminase subunit) form the PdxST PLP synthase complex; PdxK, PL kinase present in B. subtilis and *E. coli*; PdxY, PL kinase present in E. coli. PdxK from B. subtilis has PN, PL and PM kinase activity; PdxP, PNP and PLP phosphatase from *S. meliloti*; YbhA, PLP phosphatase from E. coli; PdxI, PL reductase from *E. coli*

The term vitamin B6 collectively designates the vitamers pyridoxal (PL), pyridoxamine (PM), pyridoxine (PN) and the respective phosphate esters pyridoxal-5′-phosphate (PLP), pyridoxamine-5′-phosphate (PMP) and pyridoxine-5′-phosphate (PNP) (Rosenberg 2012) (Fig. 1a). PLP is the most important B6 vitamer that is required by a variety of enzymes for catalysis (Parra et al. 2018; Hoffarth et al. 2020). Bioinformatic analyses revealed that more than 4% of the known enzymes, amongst them the majority involved in amino acid metabolism, depend on PLP (Percudani and Peracchi 2003, 2009). The importance of vitamin B6 for the physiology of the cell is highlighted by the fact that the Gram-positive model bacterium *Bacillus subtilis* is likely to have 65 PLP-dependent proteins (Richts et al. 2019). In fact, for 61 of the proteins, it has been shown that they require PLP for functioning. Moreover, in prokaryotes and eukaryotes, the activity of DNA-binding transcription factors, amongst them regulators controlling the expression of genes involved in de novo synthesis of PLP (Oka et al. 2001; Belitsky 2004a; Huq et al. 2007; Jochmann et al. 2011; El Qaidi et al. 2013; Belitsky 2014; Tramonti et al. 2015; Suvorova and Rodionov 2016; Frezzini et al. 2020). A well-studied representative of a PLP-dependent DNA-binding transcription regulator is GabR, which is responsible for regulating the expression of genes involved in the metabolism of γ-amino butyric acid (Belitsky and Sonenshein 2002; Frezzini et al. 2020). A recent proteomic approach uncovered that uncharacterized PLP-dependent enzymes can be identified even in well-studied bacteria like the Gram-positive human pathogen *Staphylococcus aureus* (Hoegl et al. 2018). Novel PLP-dependent enzymes will certainly be discovered because the number of available genome sequences is rapidly increasing.

Since animals and humans are unable to synthesise vitamin B6, these organisms have to take up vitamin B6 with their diet (Kraemer et al. 2012). Therefore, vitamin B6 is of commercial interest as a food additive and for applications in the pharmaceutical industry (Acevedo-Rocha et al. 2019). PN hypochloride, the commercial form of vitamin B6, is synthesised from PN and added usually in combination with other vitamins to food products like bakery products, cereals, baby nutrition, multivitamin juices and other beverages (Domke et al. 2005; Eggersdorfer et al. 2012). Moreover, PN is added to the food that is used for intensive animal farming to improve the yield (Johnson et al. 1950; Verbeek 1975; Eggersdorfer et al. 2012). Until today, the B6 vitamers are fully chemically synthesised using expensive and toxic chemicals (Eggersdorfer et al. 2012). Therefore, there is considerable interest in the biotech industry to develop a sustainable fermentation process for the production of vitamin B6.

This review intends to describe novel and hybrid vitamin B6 biosynthetic routes that have been identified via genetic suppressor screens using mutants of the Gram-negative and Gram-positive model bacteria *Escherichia coli* and *B. subtilis*, respectively, carrying mutations in the native genes required for synthesising the essential B6 vitamer PLP. We also discuss the potential of these pathways to serve as a starting point for the development of bacteria overproducing vitamin B6.

## De novo synthesis of the B6 vitamer PLP

As described above, PLP is the most-important B6 vitamer that is required by a variety of proteins for functioning. As yet, two naturally occurring routes for de novo synthesis of PLP are known (Mittenhuber 2001; Fitzpatrick et al. 2007; Mukherjee et al. 2011; Rosenberg et al. 2017) (Fig. 1b). The long vitamin B6 biosynthetic pathway, which involves seven enzymes and depends on the phosphor sugar deoxy-xylulose-5′-phosphate (DXP), is present in α- and γ-proteobacteria and has been extensively studied in *E. coli* (Fitzpatrick et al. 2007; Rosenberg et al. 2017). In the first part of the DXP-dependent vitamin B6 pathway, the E4P dehydrogenase Epd, the 4-phospho-erythronate (4PE) dehydrogenase PdxB and the 3-phosphoserine (3PS) aminotransferase SerC convert E4P and glutamate to 4-hydroxy-threonine-phosphate (4HTP) (Zhao et al. 1995; Drewke et al. 1996; Tazoe et al. 2006; Rudolph et al. 2010). SerC is also present in organisms that synthesise PLP via the DXP-independent vitamin B6 pathway because the enzyme is essential for serine biosynthesis (Sakai et al. 2002a, 2002b; Lam and Winkler 1990). In the second part of the DXP-dependent pathway, the 4HTP dehydrogenase PdxA oxidises 4PHT to 2-amino-3-oxo-4-(phosphohydroxy)-butyric acid, which is spontaneously decarboxylated to 1-amino-propan-2-one-phosphate (APP) (Cane et al. 1998). The PNP synthase PdxJ converts APP and DXP to PNP (Cane et al. 1999). The final reaction, yielding the B6 vitamer PLP, is catalysed by the PNP oxidase PdxH (Zhao and Winkler 1995).

The PdxST PLP synthase complex, which consists of 12 PdxS and 12 PdxT subunits, produces vitamin B6 independent of DXP (Belitsky 2004b; Raschle et al. 2005; Burns et al. 2005). The glutaminase subunit PdxT converts glutamine to glutamate and ammonia of which the latter is delivered to the PdxS PLP synthase subunit in the PdxST enzyme complex via a transient channel (Belitsky 2004b; Strohmeier et al. 2006). Since PdxS is active as a triose and pentose isomerase, the enzyme is capable of converting ammonia with either ribulose-5-phosphate and glyceraldehyde-3-phosphate or ribose-5-phosphate and dihydroxyacetone phosphate to the B6 vitamer PLP (Burns et al. 2005). The individual subunits and the PdxST enzyme complex have been well studied, both biochemically and structurally (Bauer et al. 2004; Raschle et al. 2005; Zhu et al. 2005; Strohmeier et al. 2006; Guédez et al. 2012; Smith et al. 2015; Ullah et al. 2020). The DXP-independent vitamin B6 pathway is present in archaea, bacteria, fungi, plants, Plasmodium and in some sponges (Seack et al. 2001; Ehrenshaft and Daub 2001; Fitzpatrick et al. 2007; Guédez et al. 2012). The DXP-independent vitamin B6 pathway is phylogenetically older because in silico analyses revealed that it was originally also present in α- and γ-proteobacteria that nowadays rely on the DXP-dependent pathway for PLP synthesis (Mittenhuber 2001; Tanaka et al. 2005). At a first glance, the DXP-dependent and DXP-independent vitamin B6 biosynthetic pathways seem to be different with respect to the number of involved enzymes and the catalytic mechanism. However, a structural comparison of the key biosynthetic enzymes PdxS and PdxJ of both pathways revealed similarities in their structure and catalytic mechanisms (Fitzpatrick et al. 2007).

### Salvage of B6 vitamers and proteins involved in vitamin B6 homeostasis

Most organisms, irrespective of whether they are able or unable to synthesise PLP de novo, possess a salvage pathway allowing the interconversion of the B6 vitamers PL, PN and PN and the respective phosphate esters PLP, PMP and PNP (Fitzpatrick et al. 2007; di Salvo et al. 2011). Those organisms carrying only the salvage pathway have to take up PN, PM or PL from the environment and phosphorylate them. Indeed, *E. coli* synthesises the vitamin B6 kinases PdxK and PdxY, which can convert PL into PLP (Yang et al. 1996, 1998). Recently, the PL reductase PdxI, a novel vitamin B6 salvage enzyme converting PL into PN, has been identified in *E. coli* (Ito and Downs 2020). It will be interesting to assess whether other bacteria are also endowed with this enzyme activity. The broadly conserved PLP-binding protein COG0325 is also involved in vitamin B6 homeostasis or metabolism (Prunetti et al. 2016; Tremino et al. 2017). The PLP-bound COG0325 protein from *E. coli*, YggS, has been structurally analysed (PDBid: 1W8G). Previous studies showed that the inactivation of the *yggS* gene in *E. coli* and *Salmonella enterica* causes pleiotropic phenotypes, amongst them the accumulation of PNP (Ito et al. 2019; Vu et al. 2020). Recently, it has been demonstrated that PNP interferes with glycine metabolism by competing with PLP for binding a subunit of the glycine cleavage system (Ito et al. 2020). However, the underlying molecular mechanism causing the YggS-dependent accumulation of PNP is still unclear. Interestingly, *B. subtilis* also contains a COG0325 protein, which is designated as YlmE and shares 33% overall sequence identify with YggS. In contrast to *E. coli*, *B. subtilis* relies on the DXP-independent pathway for PLP production. Therefore, it is tempting to assume that the COG0325 homologues are rather involved in modulating the salvage of B6 vitamers instead of controlling de novo synthesis of PLP. To conclude, even though vitamin B6 metabolism has already been well studied in bacteria, the functions of some proteins such as that of the conserved CO0325 proteins remain to be resolved.

### Attempts to overproduce PLP via the DXP-dependent and -independent pathways

Over the past years, several attempts have been made to engineer microorganisms for producing vitamin B6 (Rosenberg et al. 2017; Acevedo-Rocha et al. 2019). For instance, *E. coli* has been genetically engineered either by overexpressing the native genes of the DXP-dependent vitamin B6 or by overexpressing the *pdxST* genes from *B. subtilis* (Rosenberg et al. 2017). Moreover, natural overproducers of vitamin B6 such as *Sinorhizobium meliloti* have been isolated and genetically engineered for vitamin B6 production (Hoshino et al. 2006a, b). *B. subtilis* was also subjected to genetic modification for overproducing the B6 vitamer PN (Commichau et al. 2014). For this purpose, a non-native DXP-dependent pathway derived from *E. coli* and *S. meliloti* was implemented in *B. subtilis*. Growth experiments revealed that the pathway was fully functional because it relieved the PLP auxotrophy of a *pdxST* mutant (Commichau et al. 2014). *B. subtilis* was also engineered to convert the toxic metabolite 4-hydroxythreonine (4HT) to PN (Commichau et al. 2015; Rosenberg et al. 2016). The latter study revealed that *B. subtilis* possesses 4HT uptake systems and that the promiscuous homoserine kinase ThrB can convert 4HT to 4HTP, which is the substrate of the dehydrogenase PdxA. In both cases, the engineered bacteria produced significant amounts of PN. Moreover, the presence of PN in the fermentation broth suggests that *B. subtilis* must possess a phosphatase that is capable of dephosphorylating PNP and an export system for PN (Commichau et al. 2014, 2015). However, both enzyme activities remain to be identified in this organism. For a detailed summary of previous attempts to develop a sustainable fermentation process for the production of B6 vitamers, we ask the reader to consult the comprehensive review by Rosenberg and co-workers (Rosenberg et al. 2017). However, it can be stated that all microorganisms that have been genetically engineered so far did not exceed production levels of 10 g/L in 48 h that are required for outcompeting chemical synthesis processes (Acevedo-Rocha et al. 2019). Thus, major genetic engineering efforts are required for developing commercially relevant fermentation processes.

### Alternative metabolic routes for PLP biosynthesis in E. coli

**Fig. 2:**
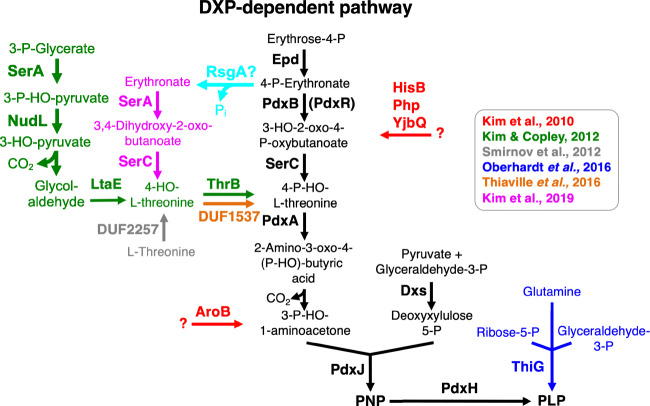
Serendipitous pathways for vitamin B6 synthesis and enzymes that feed into the DXP-dependent pathway in *E. coli*. Epd, erythrose 4-phosphate dehydrogenase; PdxB, 4-phosphoerythronatedehydrogenase; SerC, 3-phosphoserine aminotransferase; PdxA, 4-phosphohydroxy-l-threonine dehydrogenase; PdxJ, pyridoxine 5′-phosphate synthase; Dxs, 1-deoxyxylulose 5-phosphate synthase; PdxH, pyridoxine 5′-phosphate oxidase; SerA, phosphoglycerate dehydrogenase; NudL, putative NUDIX hydrolase; LtaE, l-*allo*-threonine aldolase; ThrB (and DUF1537), homoserine kinase; DUF2257, l-threonine dioxygenase; AroB, 3-dehydroquinate synthase; HisB, imidazoleglycerolphosphate dehydratase and histidinol phosphatase; Php, unknown function; YjbQ, unknown function; ThiG, thiazole synthase. RsgA is a GTPase involved in ribosome maturation in *E. coli* (Campbell and Brown 2008). RsgA shares 38% overall sequence identify with the *B. subtilis* CpgA protein, which was shown to dephosphorylate 4-phosphoerythronate (Sachla and Helmann 2019). It is tempting to speculate that RsgA is also capable of dephosphorylating 4-phosphoerythronate

Previously, it has been demonstrated that the overexpression of seven different native genes in *E. coli* (*aroA*, *hisB*, *nudL*, *pdxA*, *php*, *thrB* and *yjbQ*) relives the PLP auxotrophy of a *pdxB* mutant strain lacking the 4PE dehydrogenase PdxB (Cooper 2010; Kim et al. 2010) (Fig. 2). It is interesting to note that none of the encoded proteins acts as an enzymatic replacer for PdxB. Thus, the overproduced proteins must possess promiscuous activities that are not required for their primary function in the metabolic network of the “wild type” *E. coli* cell. Moreover, these so-called serendipitous pathways divert intermediates from other metabolic pathways and convert it to a metabolite that feeds downstream of PdxB into the DXP-dependent vitamin B6 pathway. Indeed, for one of the serendipitous pathways consisting of NudL, LtaE, SerA and ThrB it has been shown that the four enzymes connect serine to PLP biosynthesis by converting 3-phospho-glycerate to 4HTP *via* 3-phospho-hydroxypyruvate, 3-hydroxypyruvate, glycolaldehyde and 4HT (Fig. 2) (Kim et al. 2010; Kim and Copley 2012). Moreover, it has been suggested that two other serendipitous pathways consisting of at least AroA and either HisB, Php or YjbQ feed into the DXP-dependent vitamin B6 pathway upstream of SerC and PdxJ (Fig. 2). However, the reaction sequences of the latter pathways remain to be elucidated.

Recently, parallel lineages of the *E. coli pdxB* mutant strain have been evolved for up to 150 generations (Kim et al. 2019). This adaptive laboratory evolution experiment led to the identification of a novel serendipitous pathway, which consist of the three promiscuous enzymes, an unknown phosphatase required for dephosphorylating 4PE, the 3-phospho-glycerate (3PG) dehydrogenase SerA and the homoserine kinase ThrB.

The four-step serendipitous pathway feeds downstream of PdxB into the disrupted pathway and enable the bacteria to produce wild type levels of PLP (Fig. 2) (Kim et al. 2019). The detailed characterisation of the suppressor mutants revealed that some strains mutations improved the oxidation of erythronate to 3,4-dihydroxy-2-oxobutyrate by the 3PG dehydrogenase SerA, which is usually active in serine biosynthesis (Fig. 2). One mutation caused a decrease of the cellular concentrations of 3PG, the natural substrate of SerA. Another mutation decreased the cellular levels of serine, thereby preventing feedback inhibition of SerA by serine. Furthermore, several evolved *pdxB* suppressor carried mutations in the *ybhA* gene encoding a PLP phosphatase, an enzyme destroying PLP (Kuznetsova et al. 2006; Sugimoto et al. 2018; Kim et al. 2019). Thus, the so-called underground metabolism caused by promiscuous enzymes allows *E. coli* can assemble a new pathway for synthesising an essential cofactor by patching together native promiscuous enzymes (D'Ari and Casadesus 1998; Notebaart et al. 2014, 2018; Rosenberg and Commichau 2019; Copley 2020).

*E. coli* has another promiscuous enzyme, which may replace the entire DXP-dependent vitamin B6 pathway. A system-wide in silico analyses predicted that ThiG could be active in PLP synthesis in *E. coli* (Oberhardt et al. 2016). Usually, ThiG participates in the synthesis of the thiazole moiety of thiamine (Vander Horn et al. 1993). By overexpressing the *thiG* gene, it could be demonstrated that ThiG indeed restores the PLP auxotrophy of an *E. coli pdxB* mutant (Oberhardt et al. 2016). A structural model of ThiG using the structure of the *B. subtilis* PLP synthase PdxS revealed that the proteins share the same fold and potentially overlapping residues in the active site (Oberhardt et al. 2016). However, by using *E. coli pdxH* mutant lacking the *pdxH* PNP oxidase gene, it has to be experimentally validated that ThiG fulfils the same function as the PdxS PLP synthase subunit in vivo (Fig. 2).

It has also been shown that members of the DUF2257 and DUF1537 protein families can convert threonine to 4HT and 4HT to 4HTP, respectively, of which the latter is the substrate of the 4HTP dehydrogenase PdxA in the DXP-dependent vitamin B6 pathway (Figs. 1 and 2) (Smirnov et al. 2012; Thiaville et al. 2016). Another study uncovered that the DUF1537 kinase family enzymes are rather involved in the catabolism of four-carbon acid sugars such as d-erythronate and l-threonate (Zhang et al. 2016). However, the promiscuous activity of the members of the DUF2257 and DUF1537 protein families could serve as a starting point for enhancing the dioxygenase and 4HT kinase activities of the enzymes by directed evolution. These enzyme variants could be interesting to enhance the production of PNP or PLP via serendipitous pathways, which feed into the DXP-dependent vitamin B6 upstream of PdxA in *E. coli.* Indeed, in many cases, novel enzymes for practical applications are generated by directed evolution, usually starting from promiscuous functions (Yang et al. 2019; Copley 2020).

### Adaptive laboratory evolution establishes a non-native vitamin B6 pathway in B. subtilis

**Fig. 3:**
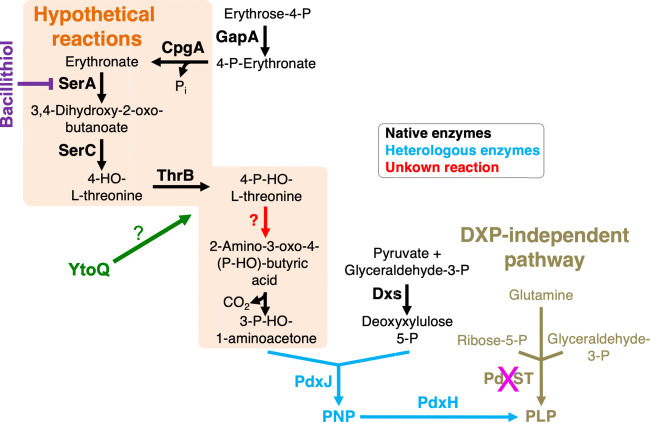
Putative serindipitous pathways for vitamin B6 synthesis in *B. subtilis*. GapA, glyceraldehyde 3-phosphate dehydrogenase; CpgA, phosphatase; SerA, phosphoglycerate dehydrogenase; SerC, 3-phosphoserine aminotransferase; ThrB, homoserine kinase; PdxJ, pyridoxine 5′-phosphate synthase; Dxs, 1-deoxyxylulose 5-phosphate synthase; PdxH, pyridoxine 5′-phosphate oxidase; PdxS (PLP synthase subunit) and PdxT (glutaminase subunit) form the PdxST PLP synthase complex; YtoQ, a protein of unknown function

As described above, *B. subtilis* relies on the PdxST PLP enzyme complex for the synthesis of the B6 vitamer PLP (Belitsky 2004b). Recently, it has assessed whether *B. subtilis* has the potential to evolve a non-native DXP-dependent vitamin B6 pathway consisting of parts of the pathway (Rosenberg et al. 2018). For this purpose, the genes encoding the enzymes of the DXP-dependent vitamin B6 pathway from *E. coli* were introduced step by step starting with the last gene of the pathway in a *B. subtilis* Δ*pdxST* mutant strain. Surprisingly, the *B. subtilis* Δ*pdxST* mutant carrying only the *pdxJ* and *pdxH* genes encoding the PNP synthase PdxJ and the PNP oxidase PdxH, respectively, formed suppressor mutants in the absence of exogenous PL (Rosenberg et al. 2018). Genome sequencing of the suppressor mutants uncovered that all strains had inactivated genes involved in the biosynthesis of bacillithiol. bacillithiol is a thiol compound, which is involved in maintaining the cellular redox balance and in the resistance to the antibiotic fosfomycin (Newton et al. 2009; Gaballa et al. 2010). It has been shown that bacillithiol may substitute for glutathione, which is a common intracellular thiol in eukaryotes and some bacteria (Newton et al. 2009). The reason why the loss of bacillithiol biosynthesis allows a *B. subtilis* Δ*pdxST* mutant carrying the *pdxJ* and *pdxH* genes to employ the DXP-dependent vitamin B6 pathway for PLP synthesis remains to be resolved (see below). However, independent lineages of the *B. subtilis* suppressor mutants were subjected to an adaptive laboratory evolution experiment to isolate variants with enhanced growth rates (Rosenberg et al. 2018). After several passages, faster-growing variants could indeed be isolated. The following genome sequencing analyses revealed that the overexpression of the *ytoQ* gene of unknown function is required to promote rapid growth of the *B. subtilis* Δ*pdxST pdxJH* mutants lacking the bacillithiol biosynthetic genes (Rosenberg et al. 2018). Thus, only two genes, encoding the enzymes that catalyse the last steps in the non-native vitamin B6 pathway, and two genomic alterations are sufficient to restore growth to wild type levels (Fig. 3). Despite the fact that the novel vitamin B6 pathway has to be fully characterised, the study shows that the underground metabolism existing in *B. subtilis* facilitates the emergence of a pathway for synthesis of the essential cofactor PLP using parts of a non-native metabolic pathway (Rosenberg et al. 2018; Rosenberg and Commichau 2019).

How could the new vitamin B6 pathway be structured? Certainly, there must be intermediates either generated by non-enzymatic catalysis of by promiscuous enzymes that feed into the truncated non-native pathway to allow synthesis of PLP. Similar to the serendipitous pathway, which connects serine biosynthesis with PLP synthesis in *E. coli*, the novel vitamin B6 pathway in the evolved *B. subtilis* Δ*pdxST pdxJH* suppressor mutants could also contain enzymes of the serine metabolic pathway (Fig. 3). Recently, it has been observed that the CpgA protein, which was previously known to be required for ribosome maturation, acts as a phosphatase dephosphorylating the toxic metabolite 4PE that inhibits the 6-phosphogluconate dehydrogenase GndA of the pentose phosphate pathway in *B. subtilis* (Sachla and Helmann 2019). As described above, 4PE is the natural substrate of PdxB of the DXP-dependent pathway in *E. coli* (Fig. 1b). Thus, α- and γ-proteobacteria either keep the cellular concentration of 4PE low by its rapid conversion via PdxB or the bacteria contain 6-phosphogluconate dehydrogenase variants that are insensitive to 4PE. However, *B. subtilis* produces an intermediate of the DXP-dependent vitamin B6 pathway that could serve as a precursor for the PLP synthesis of the truncated pathway that was implemented in the Δ*pdxST* mutant. Similar to *E. coli*, the generated erythronate could be converted via 3,4-dihydroxy-2-oxobutyrate and 4HT to 4HTP by the promiscuous activities of the 3PG dehydrogenase SerA, the serine aminotransferase SerC and the homoserine kinase ThrB (Fig. 3). This hypothesis is likely to be correct because it has been shown that the native SerC and ThrB enzymes were shown to be active in a complete non-native DXP-dependent vitamin B6 pathway and ThrB is able to convert 4HT to 4HTP, (Commichau et al. 2014, 2015). The remaining enzyme activity that would be missing would be the activity of PdxA, which does not exist in *B. subtilis*. However, several native enzymes are present in *B. subtilis* that could be part of the novel serendipitous DXP-dependent vitamin B6 pathway (Fig. 3). While it is rather unclear how the YtoQ protein of unknown function enables the evolved *B. subtilis* to employ the PdxH and PdxJ enzyme to produce PLP, the lack of bacillithiol could be beneficial because the thiol was shown to inactivate the 3PG dehydrogenase SerA by binding to the cysteine residue 410 in the active site (Chi et al. 2011; Chi et al. 2013) Thus, the loss of bacillithiol biosynthesis could enhance the promiscuous activity of SerA in the bacteria. However, this idea has to be experimentally validated.

## Conclusion and future perspectives

Previous attempts to engineer bacteria, including the Gram-negative and Gram-positive model bacteria *E. coli* and *B. subtilis*, for overproducing the B6 vitamers PN and PL were unsuccessful. In fact, major genetic engineering efforts seem to be required for developing fermentation processes that could outcompete the chemical synthesis of vitamin B6. Moreover, the suppressor analyses using *E. coli* and *B. subtilis* mutants carrying mutations in the native pathways or heterologous genes uncovered novel routes for PLP biosynthesis consisting of promiscuous enzymes and enzymes that are already involved in vitamin B6 biosynthesis. These serendipitous pathways could serve as a promising starting point for engineering the bacteria for overproducing vitamin B6 at commercially attractive levels.
